# *Notes from the Field:* Congenital Rubella Syndrome — Florida, 2025

**DOI:** 10.15585/mmwr.mm7508a2

**Published:** 2026-03-05

**Authors:** Mohammad Alak, Manuel Taffanelli Latorre, Patricia Morrill Foster, Jennifer Armstrong, Fatma Levent, Min-Hsin Chen, Timothy J. Doyle

**Affiliations:** ^1^Florida Department of Health; ^2^AdventHealth for Children, AdventHealth Medical Group, Orlando, Florida; ^3^Division of Viral Diseases, National Center for Immunization and Respiratory Diseases, CDC; ^4^Career Epidemiology Field Officer Program, Division of State and Local Readiness, Office of Readiness and Response, CDC.

**Summary**

**What is already known about this topic?**

Rubella infection during early pregnancy can result in miscarriage, fetal death, and characteristic birth defects, referred to as congenital rubella syndrome (CRS). Although rubella was declared eliminated from the United States in 2004, the disease remains a leading cause of vaccine-preventable birth defects worldwide.

**What is added by this report?**

An infant with CRS was born to a mother from a country that had not introduced rubella vaccine. The mother was likely infected during the first trimester of pregnancy, during travel to her home country. The infant had characteristic features of CRS at birth.

**What are the implications for public health practice?**

Women of reproductive age (15–49 years) without documented rubella immunity should be offered a rubella-containing vaccine before pregnancy. Clinicians should maintain awareness about rubella, especially among patients who develop a febrile rash illness after travel to regions where rubella is endemic.

In July 2025, a Florida hospital notified the Florida Department of Health (FDOH) of a case of suspected congenital rubella syndrome (CRS) in a male infant aged 6 days. The infant, born at 40 weeks’ gestation, was small for gestational age (SGA)* and had microcephaly. During the first day of life, he developed respiratory distress, cyanosis, thrombocytopenia, and a generalized rash and was admitted to the birth hospital’s neonatal intensive care unit (NICU), where a congenital heart defect (patent ductus arteriosus [PDA]), cataracts, and hearing defects were also identified. Serology testing on day 4 of life detected antirubella immunoglobulin (Ig) M antibodies. Nasopharyngeal swabs collected on day 6 of life were sent to the state’s public health laboratory, where rubella virus was identified by polymerase chain reaction, confirming the diagnosis of CRS. This activity was reviewed by CDC, deemed not research, and was conducted consistent with applicable federal law and CDC policy.^†^

## Investigation and Outcomes

### Mother’s Vaccination Status and Illness

The infant’s mother, a South African citizen aged 23 years, had lived in Florida since 2023. She reported that she had received all recommended childhood vaccinations in South Africa; however, because South Africa did not include rubella-containing vaccine (RCV) in the routine childhood immunization schedule until 2024, she was presumably not vaccinated against rubella. She visited South Africa during June 2024 and returned to Florida on September 25. On October 12, she was examined at an urgent care center with cough, nasal congestion, cervical lymphadenopathy, arthralgias, myalgias, and a rash. Rubella was not suspected, and she received a diagnosis of an unspecified viral illness.

### Pregnancy and Prenatal Care

Pregnancy was confirmed 1 month later, on November 11; a follow-up obstetric visit on November 26 estimated the gestational age to be 9 weeks, 4 days, based on the patient’s most recent menstrual period. On December 11, maternal prenatal screening demonstrated the presence of antibodies to rubella virus, providing evidence of previous infection or vaccination. During the patient’s pregnancy, she received adequate prenatal care with multiple prenatal visits and fetal ultrasound examinations; at the 20-week ultrasound, the fetus was noted to be SGA.

### Infant’s Birth and Hospital Course

When the infant was born, providers identified a constellation of signs associated with CRS, including SGA, microcephaly, rash, cataracts, and PDA. The child was immediately placed on contact precautions and admitted to the NICU, where he underwent serologic and virologic testing to confirm the diagnosis of CRS. On the 12th day of life, he was transferred to another facility for advanced NICU care and surgical repair of the PDA. He underwent extensive evaluation and was discharged home after 40 days with referrals for specialist follow-up care. Based on findings from the investigation, the mother was most likely infected with rubella virus during the first 3 weeks of pregnancy. Genotyping by CDC of the isolate obtained from the infant identified rubella virus genotype 2B, with sequences closely related to strains circulating in South Africa during 2024 (Global Measles and Rubella Laboratory Network, Rubella Virus Nucleotide Surveillance, unpublished data, 2024).

Children born with CRS are considered infectious until age 12 months, or until they receive two negative rubella virus polymerase chain reaction test results from samples collected 1 month apart (*1*). FDOH staff members contacted outpatient care providers regarding guidance on contact precautions. On October 28, testing at the FDOH laboratory confirmed that the child was no longer infectious. Contact tracing identified 22 hospital staff members who had had close contact with the child, all of whom had evidence of immunity (positive rubella antibody titers or documentation of rubella vaccination).

## Preliminary Conclusions and Actions

After an incubation period of 12–23 days, symptomatic infection with rubella virus results in a mild febrile rash illness; 25%–50% of infections are asymptomatic (*1*). However, infection during pregnancy, particularly during the first trimester, can result in CRS and is a leading cause of vaccine-preventable birth defects worldwide (*2*). During 2004, rubella and CRS were declared eliminated from the United States, although travel-associated infections and importations occur (*3*). Despite substantial progress toward global elimination in the previous decade (*2*,*4*), 16 countries do not include RCV in their routine childhood immunization schedule.^§^ Rubella remains endemic in South Africa, where a large outbreak (approximately 10,000 cases) occurred during 2024 (Measles/Rubella Dashboard | National Institute for Communicable Diseases). CRS is preventable through vaccination. Women of reproductive age (15–49 years) who do not have documentation of receipt of RCV (e.g., measles, mumps, and rubella vaccine) or other evidence of rubella immunity should be offered rubella vaccination before pregnancy (*1*). Clinicians should consider rubella among persons without evidence of rubella immunity who are evaluated for febrile rash illness, especially after travel to regions where rubella is endemic.

## Footnotes

* Birth weight of 6.48 lbs (2,940 g); a full-term male infant has a median birth weight of approximately 7.72 lbs (3,500 g).

† 45 C.F.R. part 46.102(l)(2), 21 C.F.R. part 56; 42 U.S.C. Sect. 241(d); 5 U.S.C. Sect. 552a; 44 U.S.C. Sect. 3501 et seq.

§ As of December 31, 2025, the following 16 countries did not include RCV in the routine childhood immunization schedule: Afghanistan, Central African Republic, Chad, Democratic Republic of the Congo, Djibouti, Equatorial Guinea, Ethiopia, Gabon, Guinea, Guinea-Bissau, Liberia, Madagascar, Niger, Nigeria, Somalia, and South Sudan. Mali, South Africa, and Sudan have added RCV since 2024.
